# Asthma control among treated US asthma patients in Practice Fusion’s electronic medical record research database

**DOI:** 10.1038/s41533-023-00338-7

**Published:** 2023-04-27

**Authors:** Jonathan Davitte, Bailey DeBarmore, David Hinds, Shiyuan Zhang, Jessica Chao, Leah Sansbury

**Affiliations:** 1 Value Evidence and Outcomes Data, Methods, and Analytics, GSK, Collegeville, PA USA; 2 Real World Evidence and Epidemiology, GSK, Collegeville, PA USA; 3 Value Evidence and Outcomes, GSK, Collegeville, PA USA

**Keywords:** Asthma, Asthma

## Abstract

This study investigated burden of ‘not well-controlled’ asthma, overall and by Global Initiative for Asthma (GINA) Step, among treated asthma patients in Practice Fusion’s research database. Asthma control (Asthma Control Test [ACT]) was stratified by GINA Step; prevalence ratios were estimated using Poisson regression with robust variance controlled for confounders. ACT scores ≤19 reflect not well-controlled; >19 reflect ‘well-controlled’ asthma. Of 15,579 patients, 30% had not well-controlled asthma at index date. The proportion of patients with not well-controlled asthma increased from GINA Step 1 (29%) to Step 5 (45%). Compared with Step 1, the proportion of patients with not well-controlled asthma was 0.87-times lower in Step 2, 1.10-times greater in Step 4, and 1.37-times greater in Step 5. Results suggest that despite available treatments, patients remain symptomatic across GINA Steps in real-world primary care and specialist outpatient practices, with incremental disease burden and unmet medical need in these populations.

## Introduction

Asthma is a chronic, heterogenous disease, usually characterized by chronic airway inflammation and defined by a history of respiratory symptoms including wheeze, shortness of breath, chest tightness and cough that varies both over time and in intensity together with variable expiratory airflow limitation^1^. Asthma affects 1–18% of the population across different countries^1^. In the United States (US) alone, there were an estimated 25.1 million individuals living with asthma in 2019^2^. Asthma control is defined as the degree to which asthma manifestations, such as symptoms, reliever use, lung function and exacerbations, are reduced or removed by treatment^3^, and has a major impact on patient outcomes; poor control of asthma symptoms substantially impairs health-related quality of life and is strongly associated with an increased risk of future asthma exacerbations^4–8^. The contribution of asthma severity to patient outcomes is also important to consider, as more severe forms of the disease are associated with greater symptom burden and higher asthma-related healthcare costs^9–11^. Asthma severity is determined by the intensity of treatment required to maintain good control, with more severe and difficult-to-treat asthma requiring higher dosages or supplemental treatments^3^.

The Global Initiative for Asthma (GINA) report recommends that asthma symptom control should be assessed at every opportunity, including during routine prescribing or dispensing, via direct questioning regarding symptoms and instruments designed to assess asthma control^1^. While several patient-reported instruments are available to assess asthma control, recording and integration into electronic health records (EHR) and other real-world data sources as part of routine clinical practice is limited. Consequently, while these real-world data sources contain rich data for recording asthma diagnoses and describing asthma treatment (e.g., prescriptions, claims), they have limited ability to describe asthma symptom control based on validated instruments.

In 2015, the Practice Fusion Electronic Medical Record (EMR) database integrated the Asthma Control Test (ACT) into their platform. The ACT is a patient-reported measure commonly used to distinguish different levels of symptom control by evaluating the frequency of shortness of breath and general asthma symptoms, use of rescue medications, the effect of asthma on daily functioning, and overall self-assessment of asthma control^12^. Practice Fusion, a free EMR platform, generates a notification for clinicians to consider administering the ACT or the childhood ACT (for children aged 4–11 years), whenever a patient with asthma visits the office.

The GINA report recommends that once asthma treatment has been started, ongoing decisions should be based on regular patient assessments and adjustment of treatment^1^. The asthma treatment paradigm involves five ‘treatment steps’, where asthma treatment is adjusted based on changes in asthma control status. The Steps outlined in the GINA 2019 report correspond to asthma severity: mild asthma is controlled with Step 1 or 2 treatment (as-needed controller medication alone or with low-intensity maintenance controller treatment); moderate asthma is controlled with Step 3 treatment (e.g., low-dose inhaled corticosteroids/long-acting b2 agonist [ICS/LABA]); and severe asthma requires Step 4 or 5 treatment (high-dose ICS/LABA or add-on treatments) to prevent it from becoming uncontrolled or remains uncontrolled despite this treatment^13,14^. Clinicians may recommend stepping up or stepping down asthma treatment to improve asthma control.

This study investigated the burden of not well-controlled asthma both overall, and by GINA treatment step (GINA Step), among the treated asthma patient population in Practice Fusion’s research database. While many real-world data sources allow for the investigation of asthma treatment status and patterns, along with indicators of asthma control, the absence of data from patient-reported tools in secondary sources inhibits the ability of researchers to understand the burden of not well-controlled asthma outside of clinical trials or other research settings. The Practice Fusion research database, with the integrated ACT tool, is uniquely positioned to describe asthma control among the treated asthma population as it exists in real-world primary care and specialist outpatient practices.

## Methods

### Study population

**Fig. 1:**
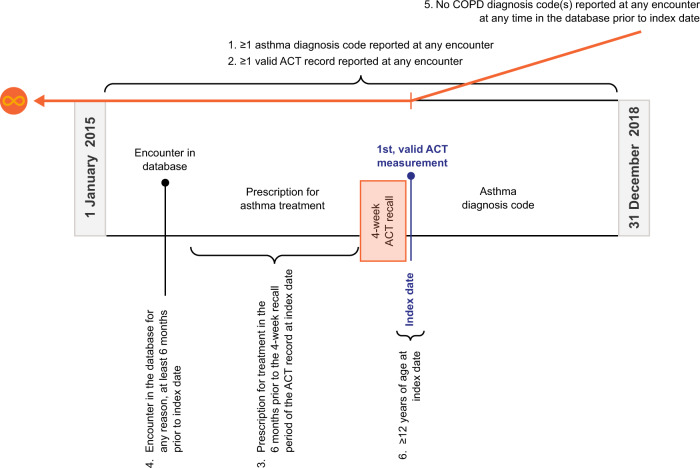
Study design. ACT asthma control test; Chronic obstructive pulmonary disease.

A retrospective cohort was established which included patients with asthma and a valid ACT measurement in Practice Fusion’s EMR database between January 1, 2015 and December 31, 2018, and with at least 1 prescription for any asthma treatment in the 6 months prior to the 4-week recall period of their first valid ACT measurement. The date of a patient’s first valid ACT measurement was defined as their index date. Patients were required to have activity in the database, defined as an encounter in the database for any reason, at least 6 months (182 days) prior to their index date. In addition, patients were excluded from our sample if they had ≥1 chronic obstructive pulmonary disease diagnosis code(s) reported at any time on or before their index date or had a missing value for their calendar year of birth (Fig. 1).

### Data source

Practice Fusion is a cloud-based connected health platform used in 30,000 healthcare practices with 8% market share among small practices (1–3 physicians) in the US, is linked with 90% of US pharmacies, and 600 laboratory and imaging entities. Practices were included in Practice Fusion’s research database if they met any of the following criteria: over 13,000 chart pulls; 1 or more providers with a verified National Provider Identifier and over 500 chart pulls; sent 500 or more electronic prescriptions; sent 500 or more laboratory orders. Practices were excluded if they were used by Practice Fusion for testing and production purposes, did not have at least one Doctor of Medicine, or were located outside the US. Practice Fusion’s research database contained patient-level data on demographics, office visits, insurance, allergies, vitals, medications, laboratory tests, diagnoses, prescriptions, and immunizations. As of December 2018, the cut of Practice Fusion’s research database contained data for 1.9 million asthma patients with ≥1 ICD-9 493.xx, ICD-10 J45.xx, or SNOMED-CT CTV3 H33xx diagnosis codes between 2007 and 2018 with patients across all 50 US states.

The database is certified as statistically de-identified through the removal of all personally identifiable indicators, transformation of dates, generalization of certain demographic and geographic information, standardization of free text and other sensitive fields, and substitution of patient- and provider-related unique identifiers with random values.

### Asthma control

The ACT is comprised of five questions, each item response is captured on a 5-point scale (where 1 is the worst scenario and 5 is the best) utilizing a 4-week recall period. ACT scores range from 5 (poor control of asthma) to 25 (complete control of asthma) with higher scores reflecting greater asthma control. ACT scores ≤19 reflect not well-controlled asthma while ACT scores >19 reflect well-controlled asthma^15^.

Beginning in 2015, Practice Fusion implemented a clinical decision support program that notified providers that an ACT should be conducted when a patient with asthma missing symptom assessments visited them. While the notification indicated that an ACT should be completed, the system did not require clinicians to complete and/or record the ACT results.

We defined a valid ACT as: (1) having complete responses for all 5 questions; (2) not occurring on the same date as another ACT measurement for the same patient; and (3) not occurring within 28 days of another ACT measurement for the same patient. Scores that reflect asthma control as measured by the ACT cannot be calculated if any of the 5 questions are missing responses. The rationale behind this 28-day time gap is that the ACT reflects a 4-week recall period; if two ACT scores are measured on the same day or within 28 days of each other, it is impossible to determine which of these indicate the correct measurement of asthma control.

### GINA step

GINA Step was assessed based on the medications prescribed during the 6-month period prior to the 4-week recall period of patients’ ACT record at index date. Asthma treatment was defined as one of the following medications: short-acting β_2_-agonists (SABA), short-acting muscarinic antagonist (SAMA), inhaled corticosteroids (ICS), ICS and long-acting β_2_-agonist (ICS/LABA) combination products, leukotriene receptor antagonist, cromolyn or nedocromil (mast cell stabilizers), methylxanthines, biologics (e.g., mepolizumab) or long-acting muscarinic antagonist. Further details on GINA Step definition and asthma treatments are in Supplementary Table 1.

Determination of a patient’s GINA Step required calculation of ICS and ICS/LABA daily doses. The Practice Fusion prescription data includes fields that were generated using MedEx, a natural language processing system which extracts medication information from clinical notes^16^. There are three MedEx-derived fields that were used for calculation of ICS daily dose: (1) frequency *(e.g., once per day*); (2) dose amount (*e.g., ‘2’ in ‘2 puffs’*); (3) dose unit (*‘puff’ in ‘2 puffs’*). For missing values of frequency, dose, or dose amount, we imputed values from the mode across each National Drug Code. We converted all ICS strength to micrograms (mcg) prior to calculating ICS daily dose^17^. ICS daily dose was calculated as: (Frequency)*(Dose amount)*(Strength). Finally, the ICS and ICS/LABA dosage levels required for GINA Step calculation were defined for each medication based on generic names or ingredients (Supplementary Table 2).

ICS/LABA includes fixed-dose ICS/LABA combination medications and ‘open’ ICS/LABA combinations. For patients that had individual ICS and LABA prescriptions, we considered them as ‘open’ ICS/LABA combinations only if the ICS medication and LABA medication were prescribed within 30 days of each other. Patients with separate ICS and LABA prescriptions more than 30 days apart were considered as ‘ICS only’ in the GINA Step calculation. For patients that had multiple ICS or ICS/LABA prescriptions in the eligible period, we used only the prescription(s) that were closest to the patient’s index date for calculation of the ICS daily dose.

GINA steps were defined according to GINA asthma treatment guidelines in 2018^17^. The GINA 2019 treatment guidelines include the addition of as-needed low-dose ICS-formoterol for Step 1^13^. Given that this additional criteria for Step 1 did not align with our observation period, we chose to define GINA Step according to the guidelines clinicians would have followed at the time they prescribed asthma medications in our study. We assumed that any oral corticosteroid (OCS) use was not used continuously (e.g., supply ≤28 days) by the patient and thus had no impact on GINA Steps. This decision was made given the difficulty in calculating a consistent day supply for OCS from the Practice Fusion prescription data. Treatment with SABA, SABA-SAMA, or SAMA was classified simply as SABA. Treatment with SABA only was defined as GINA Step 1. However, SABA use was allowed in all other steps. All individuals that were missing key information required for the GINA Step determination or had combinations of prescriptions that did not clearly meet definitions for a GINA Step were classified as ‘Undefined’.

### Covariates

Age in years was calculated as the difference between the calendar year of a patient’s index date and their birth year. Ethnicity was defined as ‘Hispanic’, ‘Non-Hispanic’ or ‘Missing’. Race was defined as ‘White’, ‘Black/African American’, ‘Other’ and ‘Unknown’. ‘Unknown’ race was assigned to individuals that had conflicting responses for race at any time in the database (e.g., patients may have multiple race information) or did not have any documentation of race in the Practice Fusion database. ‘Current’ smoking status was assigned to patients with a status of ‘current smoker’ on the smoking status record closest to their index date. ‘Former’ was assigned to patients with smoking status of ‘former smoker’ at any time on or before their index date. ‘Non-smoker’ was assigned to patients with only records of ‘non-smoker’ at any time in the database on or prior to their index date. Finally, we used the value for body mass index (BMI) in kg/m^2^ that was recorded on the individual’s index date or a prior record closest (e.g., least number of days) to the index date. Visit type was categorized according to the specialty of the provider with whom the patient had an appointment for the encounter on their index date: ‘Primary Care’ includes ‘Internal Medicine’, ‘General Medicine’, and ‘Family Medicine’; ‘Specialist’ includes ‘Allergy and Immunology’, ‘Pulmonary Disease’, and ‘Emergency Medicine’; ‘Other’ includes all other specialties.

### Statistical analysis

Descriptive frequencies both overall and by patient asthma control status at index date were calculated for each GINA Step. We used Poisson regression with robust variance to directly estimate the prevalence ratio (PR) and 95% confidence intervals (95% CI) of not well-controlled asthma by patient GINA Step at index date, adjusting for age, race, Hispanic ethnicity, smoking status, BMI, and the visit type at index date. Given that not well-controlled asthma was quite common in our study population (e.g., >10%), odds ratios derived from logistic regression would violate the rare disease assumption and consequently would overestimate the strength of associations and not approximate the relative risk^18^. However, Poisson regression models with robust variance can directly estimate the PR and are a suitable alternative to logistic regression modeling in cross-sectional studies with a dichotomous outcome^19^. The primary exposure of interest was GINA Step at index date with Step 1 as the reference group. The dependent variable (outcome) was not well-controlled asthma at index date, defined as an ACT score ≤19. We used a Directed Acyclic Graph to identify covariates for confounding control in the regression model. The final model included age (in years), BMI, race, Hispanic ethnicity, smoking status, and type of visit at index date.

### Ethics

The data used in this study are data collected from routine activity as part of patients’ interactions with the healthcare system through their provider’s medical records software. The original data collection is for administration and healthcare delivery purposes but is aggregated and deanonymized for research purposes. The analysis used fully deidentified retrospective data, and as such, this is not classified as research involving human participants as defined by 45 CFR 46.102(f) under the US Department of Health and Human Services Policy for Protection of Human Subjects (https://www.hhs.gov/ohrp/regulations-and-policy/regulations/2018-req-preamble/index.html). Therefore, institutional review board approval and informed consent were not required.

### Reporting summary

Further information on research design is available in the Nature Research Reporting Summary linked to this article.

## Results

### Overall baseline characteristics

**Fig. 2:**
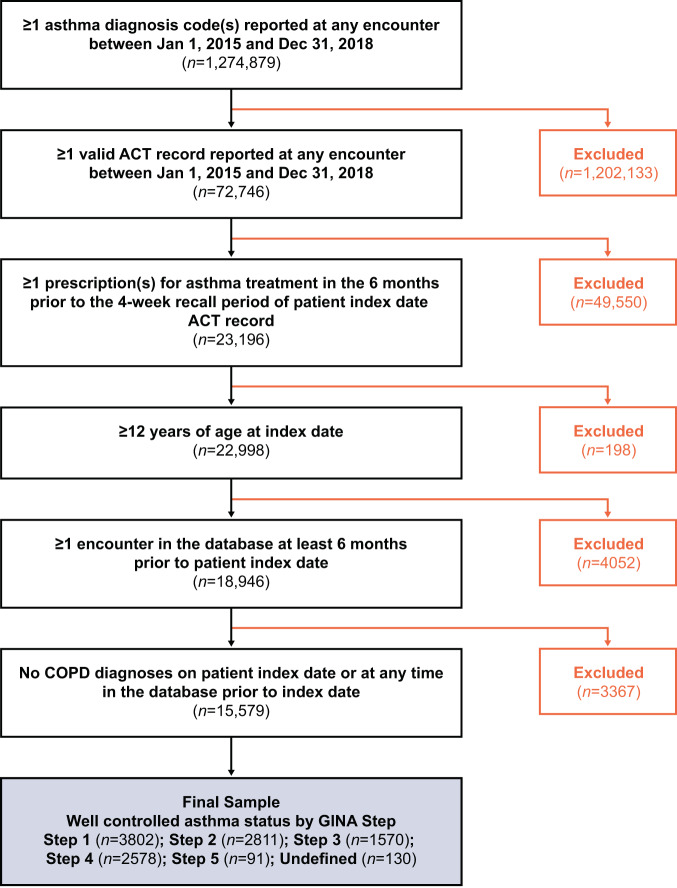
Construction of study sample. Inclusion and exclusion criteria applied to construct the final study sample. ACT asthma control test, COPD chronic obstructive pulmonary disease, GINA Global Initiative for Asthma.

Overall baseline characteristics are shown in Table 1. We identified 15,579 treated patients with asthma for our study sample after applying all inclusion and exclusion criteria (Fig. 2). Overall, the sample had a mean age of 44 years (standard deviation = 22) and was predominantly female (64%, *n* = 9995), non-Hispanic (80%, *n* = 12,489), and white (46%, *n* = 7153) (Table 1). The majority of the sample received their index ACT record at a primary care visit (55%, *n* = 8527) with 22% (*n* = 3352) receiving their index ACT at a specialist visit and 22% (*n* = 3485) at a non-primary care/non-specialist visit.

**Table 1:** Asthma control status and patient characteristics by GINA Step among the treated asthma patient population, Practice Fusion EMR, 2015–2018 (*N* = 15,579).

Characteristics	GINA step^a^						Overall (*N* = 15,579)
	Step 1 (*N* = 5374)	Step 2 (*N* = 3751)	Step 3 (*N* = 2187)	Step 4 (*N* = 3909)	Step 5 (*N* = 165)	Undefined (*N* = 193)	
Asthma control^b^, *n* (%)							
Not well-controlled	1572 (29.3)	940 (25.1)	617 (28.2)	1331 (34.0)	74 (44.8)	63 (32.6)	4597 (29.5)
Well-controlled	3802 (70.7)	2811 (74.9)	1570 (71.8)	2578 (66.0)	91 (55.2)	130 (67.4)	10982 (70.5)
Age in years							
Mean (SD)	39.4 (21.8)	44.1 (22.7)	43.9 (22.7)	49.3 (20.6)	55.9 (16.6)	54.9 (19.5)	44.0 (22.2)
Range	12.0–88.0	12.0–88.0	12.0–88.0	12.0–88.0	12.0–87.0	12.0–88.0	12.0–88.0
Sex, male, *n* (%)	1996 (37.1)	1310 (34.9)	787 (36.0)	1359 (34.8)	59 (35.8)	73 (37.8)	5584 (35.8)
Hispanic ethnicity, Yes, *n* (%)	1150 (21.4)	680 (18.1)	426 (19.5)	786 (20.1)	15 (9.1)	33 (17.1)	3090 (19.8)
Race^c^, *n* (%)							
White	2362 (44.0)	1864 (49.7)	946 (43.3)	1826 (46.7)	64 (38.8)	91 (47.2)	7153 (45.9)
African American	946 (17.6)	557 (14.8)	402 (18.4)	647 (16.6)	30 (18.2)	29 (15.0)	2611 (16.8)
Other	424 (7.9)	280 (7.5)	201 (9.2)	321 (8.2)	6 (3.6)	21 (10.9)	1253 (8.0)
Unknown	1642 (30.6)	1050 (28.0)	638 (29.2)	1115 (28.5)	65 (39.4)	52 (26.9)	4562 (29.3)
Smoking status^d^, *n* (%)							
Non-smoker	3467 (64.5)	2677 (71.4)	1482 (67.8)	2589 (66.2)	90 (54.5)	114 (59.1)	10419 (66.9)
Former smoker	628 (11.7)	452 (12.1)	307 (14.0)	602 (15.4)	34 (20.6)	39 (20.2)	2062 (13.2)
Current smoker	660 (12.3)	244 (6.5)	172 (7.9)	373 (9.5)	19 (11.5)	26 (13.5)	1494 (9.6)
Unknown	619 (11.5)	378 (10.1)	226 (10.3)	345 (8.8)	22 (13.3)	14 (7.3)	1604 (10.3)
Body mass index^e^, kg/m^2^							
Mean (SD)	31.1 (8.4)	31.1 (8.1)	31.1 (8.3)	31.9 (8.3)	33.2 (9.0)	31.0 (8.8)	31.3 (8.3)
Range	13.7–68.3	15.3–66.4	14.3–64.5	14.6–70.4	16.5–60.5	15.7–60.5	13.7–70.4
Body mass index category, *n* (%)							
Underweight	100 (1.9)	70 (1.9)	33 (1.5)	55 (1.4)	1 (0.6)	7 (3.6)	266 (1.7)
Normal	964 (17.9)	662 (17.6)	427 (19.5)	651 (16.7)	21 (12.7)	39 (20.2)	2764 (17.7)
Overweight	1179 (21.9)	853 (22.7)	474 (21.7)	938 (24.0)	44 (26.7)	49 (25.4)	3537 (22.7)
Obese	2116 (39.4)	1511 (40.3)	894 (40.9)	1921 (49.1)	90 (54.5)	85 (44.0)	6617 (42.5)
Unknown	1015 (18.9)	655 (17.5)	359 (16.4)	344 (8.8)	9 (5.5)	13 (6.7)	2395 (15.4)
Visit type, *n* (%)							
Primary care	3061 (57.0)	2064 (55.0)	1058 (48.4)	2158 (55.2)	65 (39.4)	121 (62.7)	8527 (54.7)
Specialist	570 (10.6)	820 (21.9)	585 (26.7)	1234 (31.6)	92 (55.8)	51 (26.4)	3352 (21.5)
Other	1664 (31.0)	819 (21.8)	514 (23.5)	466 (11.9)	6 (3.6)	16 (8.3)	3485 (22.4)
Unknown/missing	79 (1.5)	48 (1.3)	30 (1.4)	51 (1.3)	2 (1.2)	5 (2.6)	215 (1.4)

^a^GINA Step defined using prescriptions for asthma treatment in the 6 months prior to the 4-week recall period of patient’s index date ACT record.

^b^Asthma control defined by ACT scores: ‘Not well-controlled’ ≤19 and ‘Well-controlled’ >19.

^c^‘Unknown’ race assigned to patients that have (1) conflicting responses for race and/or (2) no entries for race recorded in the system.

^d^‘Current’ smoking status assigned to patients with a status of ‘current smoker’ on the date closest to index date. ‘Former’ assigned to patients with smoking status of ‘former smoker’ at any time before their index date. ‘Non-smoker’ assigned to patients with only records of ‘non-smoker’ at any time in the database prior to their index date.

^e^Body mass index measurement recorded on the same date or the date closest to their index date.

*ACT* asthma control test, *EMR* electronic medical record, *GINA* Global Initiative for Asthma, *SD* standard deviation.

### Baseline demographics and characteristics by GINA step

Individuals in GINA Step 1 were younger than the other GINA Step groups: mean 39.4 years for Step 1 compared with 44–56 years for Steps 2–5 and ‘Undefined’. Patients in the Step 5 GINA group were the oldest (mean 56 years). The sex composition was similar across all GINA Steps with males comprising 35 to 38% of each GINA Step group (Table 1).

Approximately 20% of patients in GINA Steps 1–4 and Undefined groups were of Hispanic Ethnicity. By comparison, GINA Step 5 had substantially fewer individuals of Hispanic Ethnicity (9.1%, *n* = 15). The racial composition for white and African Americans across GINA Step groups (1–5) was relatively similar with 39–50% and 15–18%, respectively. While proportion of patients self-identifying as ‘Other’ and ‘Unknown’ race were similar for GINA Steps 1–4 and Undefined groups, GINA Step 5 had a much smaller proportion of patients identifying as ‘Other’ race and a greater proportion of those with ‘Unknown’ race.

While GINA Steps 1–4 groups had similar proportions of non-smokers (65–71%) and former smokers (12–15%), Step 5 and ‘Undefined’ groups had fewer non-smokers (55%, *n* = 90 and 59%, *n* = 114, respectively) and more former smokers (21%, *n* = 34 and 20%, *n* = 39, respectively). The greatest proportion of current smokers was observed in the ‘Undefined’ GINA Step group (14%, *n* = 26) followed by Step 1 (12%, *n* = 660), Step 5 (12%, *n* = 19), Step 4 (10%, *n* = 373), Step 3 (8%, *n* = 172), and Step 2 (7%, *n* = 244).

The percentage of individuals with ‘Obese’ BMI increased from GINA Step 1–5 with 39% (*n* = 2116) in Step 1, 40% (*n* = 1511) in Step 2, 41% (*n* = 894) in Step 3, 49% (*n* = 1921) in Step 4, and 55% (*n* = 90) in Step 5.

Similar proportions of ACT records at index date were recorded in primary care visits for GINA Steps 1–4 (48%–57.0%). However, relatively few individuals in GINA Step 1 received their index date ACT at a specialist visit (1%, *n* = 570) compared with 56% (*n* = 92) for Step 5, 32% (*n* = 1234) for Step 4, 27% (*n* = 585) for Step 3, and 22% (*n* = 820) for Step 2.

### Asthma control overall and by GINA step

At index date, 30% (*n* = 4597) of individuals had not well-controlled asthma compared with 71% (*n* = 10,982) with well-controlled asthma (Table 1). With respect to GINA Step, 35% (*n* = 5374) of the overall population were classified in Step 1; 24% (*n* = 3751) in Step 2; 14% (*n* = 2187) in Step 3; 25% (*n* = 3909) in Step 4; 1% (*n* = 165) in Step 5; and 1% (*n* = 193) were ‘Undefined’ GINA Step (Table 2). Distribution by GINA Step was similar for individuals with not well-controlled asthma compared with individuals with well-controlled asthma for Step 1 (34%, *n* = 1572 vs. 35%, *n* = 3802), Step 3 (13%, *n* = 617 vs. 14%, *n* = 1570), and ‘Undefined’ (1%, *n* = 63 vs. 1%, *n* = 130). However, fewer individuals with not well-controlled asthma were classified in GINA Step 2 compared with individuals with well-controlled asthma: 20% (*n* = 940) and 26% (*n* = 2811), respectively. By comparison, a larger proportion of individuals with not well-controlled asthma were classified as GINA Step 4 and Step 5 compared with individuals with well-controlled asthma; 29% (*n* = 1331) of individuals with not well-controlled asthma were in Step 4 compared with 23% (*n* = 2578) with well-controlled asthma, and in Step 5 the proportions were 2% (*n* = 74) and 1% (*n* = 91), respectively (Table 2).

**Table 2:** GINA Step, patient characteristics and provider characteristics by asthma control status among the treated asthma patient population, Practice Fusion EMR, 2015–2018 (*N* = 15,579).

	Asthma control status^a^		Overall (*N* = 15,579)
	Well-controlled (*N* = 10,982)	Not well-controlled (*N* = 4597)	
GINA step^b^			
Step 1	3802 (34.6%)	1572 (34.2%)	5374 (34.5%)
Step 2	2811 (25.6%)	940 (20.4%)	3751 (24.1%)
Step 3	1570 (14.3%)	617 (13.4%)	2187 (14.0%)
Step 4	2578 (23.5%)	1331 (29.0%)	3909 (25.1%)
Step 5	91 (0.8%)	74 (1.6%)	165 (1.1%)
Undefined	130 (1.2%)	63 (1.4%)	193 (1.2%)
Age			
Mean (SD)	43.1 (22.7)	46.3 (20.8)	44.0 (22.2)
Range	12.0–88.0	12.0–88.0	12.0–88.0
Sex, male, *n* (%)	4099 (37.3)	1485 (32.3)	5584 (35.8)
Hispanic ethnicity, Yes, *n* (%)	2185 (19.9)	905 (19.7)	3090 (19.8)
Race^c^, *n* (%)			
White	5154 (46.9)	1999 (43.5)	7153 (45.9)
African American	1784 (16.2)	827 (18.0)	2611 (16.8)
Other	927 (8.4)	326 (7.1)	1253 (8.0)
Unknown	3117 (28.4)	1445 (31.4)	4562 (29.3)
Smoking status^d^, *n* (%)			
Non-smoker	7547 (68.7)	2872 (62.5)	10,419 (66.9)
Former smoker	1389 (12.6)	673 (14.6)	2062 (13.2)
Current smoker	895 (8.1)	599 (13.0)	1494 (9.6)
Unknown	1151 (10.5)	453 (9.9)	1604 (10.3)
Body mass index^e^, kg/m^2^			
Mean (SD)	30.9 (8.1)	32.2 (8.7)	31.3 (8.3)
Range	13.7–68.3	14.6–70.4	13.7–70.4
Body mass index category, *n* (%)			
Underweight	187 (1.7)	79 (1.7)	266 (1.7)
Normal	1993 (18.1)	771 (16.8)	2764 (17.7)
Overweight	2539 (23.1)	998 (21.7)	3537 (22.7)
Obese	4385 (39.9)	2232 (48.6)	6617 (42.5)
Unknown	1878 (17.1)	517 (11.2)	2395 (15.4)
Visit type, *n* (%)			
Primary care	5795 (52.8)	2732 (59.4)	8527 (54.7)
Specialist	2358 (21.5)	994 (21.6)	3352 (21.5)
Other	2679 (24.4)	806 (17.5)	3485 (22.4)
Unknown/missing	150 (1.4)	65 (1.4)	215 (1.4)
Practice census region, *n* (%)			
Midwest	1856 (16.9)	819 (17.8)	2675 (17.2)
Northeast	2279 (20.8)	921 (20.0)	3200 (20.5)
South	4607 (42.0)	1831 (39.8)	6438 (41.3)
West	1697 (15.5)	763 (16.6)	2460 (15.8)
Unknown	543 (4.9)	263 (5.7)	806 (5.2)

*ACT* asthma control test, *EMR* electronic medical record, *GINA* Global Initiative for Asthma, *SD* standard deviation.

^a^Asthma control defined by ACT scores: ‘Not well-controlled’ ≤19 and ‘Well-controlled’ >19.

^b^GINA Step defined using prescriptions for asthma treatment in the 6 months prior to the 4-week recall period of patient’s index date ACT record.

^c^‘Unknown’ race assigned to patients that have (1) conflicting responses for race and/or (2) no entries for race recorded in the system.

^d^‘Current’ smoking status assigned to patients with a status of ‘current smoker’ on the date closest to index date. ‘Former’ assigned to patients with smoking status of ‘former smoker’ at any time before their index date. ‘Non-smoker’ assigned to patients with only records of ‘non-smoker’ at any time in the database prior to their index date.

^e^Body mass index measurement recorded on the same date or the date closest to their index date.

**Fig. 3:**
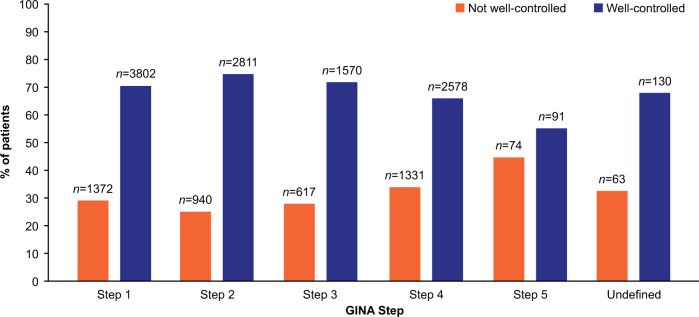
Asthma control status of patients by GINA Step at index date. Percentage and number of individuals with ‘Not well-controlled’ and ‘Well-controlled’ asthma. GINA Global Initiative for Asthma.

Across all GINA Steps more than a quarter of individuals were classified as having not well-controlled asthma (Table 1). Figure 3 illustrates the absolute numbers and percentages of individuals with well-controlled and not well-controlled asthma at their index date by GINA Step. The largest proportion of individuals with not well-controlled asthma was within GINA Step 5 (45%, *n* = 74); although it should be noted that relatively few individuals were classified in GINA Step 5 (*n* = 165) compared with the other steps. Among GINA Steps 1–4 (which had similarly large numbers of individuals), the largest burden of not well-controlled asthma was present in GINA Step 4 (34%, *n* = 1331) followed by Step 1 (29%, *n* = 1572), Step 3 (28%, *n* = 617), and Step 2 (25%, *n* = 940). Additional information on asthma control distribution by patient and provider characteristics can be found in Table 2.

### Association between GINA step and asthma control at index date

**Fig. 4:**
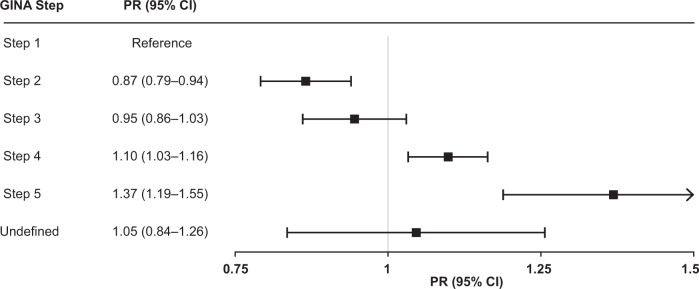
Association between GINA Step and asthma control at index date. Adjusted prevalence ratio of ‘Not well-controlled’ asthma by GINA Step among the treated asthma population. CI confidence interval, GINA Global Initiative for asthma, PR prevalence ratio.

Compared with patients in Step 1, the proportion of patients with not well-controlled asthma was 0.87 times lower among patients in Step 2 (PR: 0.87, 95% CI: 0.79–0.94), 1.10 times greater among patients in Step 4 (PR: 1.10, 95% CI: 1.03–1.16), and 1.37 times greater among patients in Step 5 (PR: 1.37, 95% CI: 1.19–1.55), after adjusting for age, race, Hispanic ethnicity, smoking status, BMI, and visit type at index date (Fig. 4). We did not observe significant differences in not well-controlled asthma among patients in Step 3 compared with Step 1 in our fully adjusted model.

## Discussion

Results from this sample of 15,579 treated patients with asthma in the US showed that despite a variety of available treatments, patients with asthma remain symptomatic across GINA Steps in real-world primary care and specialist outpatient practices. At index date, nearly one-third of our sample had not well-controlled asthma despite receiving prescriptions for asthma treatment in the 6 months prior to the 4-week recall period of their ACT record. The proportion of patients with not well-controlled asthma in our sample was lower than in previous studies under more controlled study design^20,21^. This may indicate a potential bias in patients that were administered the ACT. We observed an increasing proportion of individuals with not well-controlled asthma from GINA Step 1 to GINA Step 5 in our descriptive analysis. However, when comparing GINA Steps, our modeling results only showed significant differences in the proportion of patients with not well-controlled asthma between Steps 2, 4, and 5 compared with Step 1 after full adjustment (not Step 3). We observed significantly better asthma control in GINA Step 2 compared with GINA Step 1; while we observed significantly worse asthma symptom control in GINA Steps 4 and 5 compared with GINA Step 1. The finding that asthma control was significantly better in GINA Step 2 compared with Step 1 may also suggest that patients in the Step 1 group are misclassified and undertreated, and therefore should be assigned to a higher GINA Step.

The significantly better asthma control among patients in GINA Step 2 compared with GINA Step 1 may be attributed to the addition of a regular controller medication in addition to a reliever medication in Step 2 compared with only a reliever for Step 1. As-needed SABA with no controller was the recommendation for Step 1 in the GINA 2018 guidelines which were used during the observation period for this study^17^. However, in a major change from the 2018 recommendations, the GINA 2019 guidelines no longer recommend SABA-only treatment (without ICS) and instead recommend as-needed low-dose combination ICS-formoterol as a controller for Step 1 in addition to the reliever^13^.

We did not see a significant difference in asthma control when comparing patients in Step 3 with the Step 1 group. Steps 4 and 5 represent severe and difficult-to-treat asthma^13,14^, which likely account for the significantly higher proportions of not well-controlled asthma in these steps compared with Step 1.

There are several strengths to our study that warrant mention. Our study was able to link systematically measured asthma symptom control via the ACT and prescription information in a real-world setting. We also had sufficient prescription information and history within Practice Fusion’s EMR to calculate GINA Steps for our patient population, enabling us to describe burden of not well-controlled asthma among patients across all GINA Steps. Additionally, the use of this real-world data source allowed our study to identify a large number of patients with asthma, across the entire asthma continuum, with a mixture of asthma symptom control. Finally, we were able to demonstrate that the significant differences in asthma control at higher GINA Steps were maintained after adjusting for confounding.

Despite the variety and availability of asthma treatment, there are large numbers of patients with asthma that remain symptomatic across the treatment continuum in real-world primary care and specialist outpatient practices. Consequently, our study highlights the importance of assessing asthma symptom control at every opportunity; and evaluating treatment response, inhaler technique, adherence, and environmental exposures for patients with symptomatic asthma. There are several limitations to our study. The EHR data is intended for clinical decision making, not research. Thus, in using routinely collected healthcare data for research we must recognize the limitations in data quality. In addition, the Practice Fusion asthma patient population is an open cohort, in which patients may enter or leave care at any time. As such, while investigating changes in GINA Step over time may provide added insight into patients’ asthma control status, the current study was limited by the data available in the Practice Fusion database.

Moreover, given that Practice Fusion is used primarily by smaller outpatient primary and specialist care offices in the US, it is likely that the asthma patient population in the Practice Fusion research database will be systematically different from the overall US asthma population which includes patients seen in large practices, inpatient facilities, or other healthcare settings. Indeed, patients in this study population were primarily of White or Unknown race, making it challenging to assess the impact of variables such as socioeconomic status and race/ethnicity on asthma control, which have previously been linked to asthma-related health outcomes^22–25^. Additionally, as the dataset does not include all levels of care (e.g., inpatient care) or data from large healthcare systems, the study population may represent patients with better control/less severe disease compared with a sample that included ACT measures from across the healthcare continuum. Further, the ACT was administered by clinicians via a clinical decision support prompt in the EHR rather than self-administered by the patient. This process leads to the possibility of reporting bias as patient answers may reflect how they would like to be perceived by the clinician rather than their actual experience.

There are also limitations in using GINA Step categorization for research purposes. Prescriptions represent the intent of the prescriber not actual medication use or adherence. We assumed that patients took their prescription medications as prescribed. We were reliant on natural language processing of prescription signature information captured in the Practice Fusion database for the calculation of ICS daily dose; which may have incorrectly specified actual frequency and/or dosage. Due to the limitations with the prescription data in the Practice Fusion EHR, we cannot account for daily OCS that is typically used in calculating GINA Step 5. We had to assume that all OCS use was less than or equal to 28 days; and, consequently had no impact on GINA Step. Furthermore, it is worth noting that the current study did not differentiate between not well-controlled and poorly controlled asthma.

Lastly, our analysis did not assess the duration at which a patient was in a GINA Step. Given the nature of the EHR data, our patient population contained a mixture of patients that: (1) recently initiated asthma treatment (new users); (2) recently modified their previous asthma treatment; and 3() had used a specific asthma treatment for an extended period and were renewing the prescription that had been working for them (prevalent users). Our analysis was not able to distinguish between these three distinct groups of patients.

## Supplementary information

Supplementary Material File

Reporting Summary

## Data Availability

Anonymized individual participant data and study documents can be requested for further research from www.clinicalstudydatarequest.com.
