# Supplementary material for: Asthma control among treated US asthma patients in Practice Fusion’s electronic medical record research database
Source: NPJ Prim Care Respir Med. 2023 Apr 27;33:17. doi: 10.1038/s41533-023-00338-7 (PMC10140050; doi:10.1038/s41533-023-00338-7)
Supplement: Supplementary file 1 — Supplementary Material File [file 41533_2023_338_MOESM1_ESM.pdf]

Asthma control among treated US asthma patients in Practice Fusion's electronic medical record research database

Jonathan Davitte, PhD, Bailey DeBarmore, MHS RDN, David Hinds, BA MHS,

Shiyuan Zhang, MSc, Jessica Chao, PhD, Leah Sansbury, PhD

Supplementary information

Supplementary Table 1. GINA Step definitions and asthma treatments[1]

GINA Step	Treatments
GINA Step 1	• SABA[2] only
GINA Step 2	• Low-dose ICS only<br>• LTRA only<br>• Low-dose Theophylline only
GINA Step 3	• Low-dose ICS-LABA<br>• Medium/High-dose ICS without an additional controller<br>• Low-dose ICS + LTRA or Theophylline
GINA Step 4	• Medium/High-dose ICS/LABA + an additional controller (excluding Tiotropium)<br>• GINA Step 3 + an additional controller
GINA Step 5	• Mepolizumab ± any others<br>• Xolair ± any others<br>• GINA Step 4 + OCS[3]<br>• Medium/High-dose ICS/LABA + Tiotropium (LAMA) ± any others
GINA Step-Undefined	• If not specified in any other steps

[1]The definitions reflect the GINA asthma treatment guidelines in 2018.

[2]Treatment with SABA, SABA-SAMA, or SAMA were classified as SABA only. SABA was allowed in all other steps.

[3]It was assumed that OCS was not used continuously by the patient and thus had no impact on the GINA Step classification.

GINA, Global Initiative for Asthma; ICS, inhaled corticosteroid; LABA, long-acting β_2-agonist; LAMA, long-acting muscarinic antagonist; LTRA, leukotriene receptor antagonist; OCS, oral corticosteroid; SABA, short-acting β_2-agonist; SAMA, short-acting muscarinic antagonist.

Supplementary Table 2. ICS and ICS/LABA dosage levels by generic names/ingredients

Generic name	ICS daily dose	ICS, ICS/LABA dosage level
Beclomethasone	≤200<br>200–400<br>>400	Low<br>Medium<br>High
Budesonide + Product name = 'Pulmicort respules'	≤500<br>500–1000<br>>1000	Low<br>Medium<br>High
Other Budesonide	≤400<br>400–800<br>>800	Low<br>Medium<br>High
Ciclesonide	≤160<br>160–320<br>>320	Low<br>Medium<br>High
Flunisolide	≤1000<br>1000–2000<br>>2000	Low<br>Medium<br>High
Fluticasone	≤250<br>250–500<br>>500	Low<br>Medium<br>High
Fluticasone furoate	Strength contains 100<br>Strength contains 200<br>Other	Low<br>Medium<br>Undefined
Mometasone	≤220<br>220–440<br>>440	Low<br>Medium<br>High
Triamcinolone acetonide	≤1000<br>1000–2000<br>>2000	Low<br>Medium<br>High

ICS, inhaled corticosteroid; LABA, long-acting β_2-agonist
