# Supplementary material for: Asthma control among treated US asthma patients in Practice Fusion’s electronic medical record research database
Source: NPJ Prim Care Respir Med. 2023 Apr 27;33:17. doi: 10.1038/s41533-023-00338-7 (PMC10140050; doi:10.1038/s41533-023-00338-7)
Supplement: Supplementary file 2 — Reporting Summary [file 41533_2023_338_MOESM2_ESM.pdf]

nature portfolio

Corresponding author(s): Shiyuan Zhang

Last updated by author(s): Nov 3, 2022

Reporting Summary

Nature Portfolio wishes to improve the reproducibility of the work that we publish. This form provides structure for consistency and transparency in reporting. For further information on Nature Portfolio policies, see our Editorial Policies and the Editorial Policy Checklist.

Statistics

For all statistical analyses, confirm that the following items are present in the figure legend, table legend, main text, or Methods section.

n/a	Confirmed	
☐	☒	The exact sample size (*n*) for each experimental group/condition, given as a discrete number and unit of measurement
☒	☐	A statement on whether measurements were taken from distinct samples or whether the same sample was measured repeatedly
☐	☒	The statistical test(s) used AND whether they are one- or two-sided<br>*Only common tests should be described solely by name; describe more complex techniques in the Methods section.*
☐	☒	A description of all covariates tested
☐	☒	A description of any assumptions or corrections, such as tests of normality and adjustment for multiple comparisons
☐	☒	A full description of the statistical parameters including central tendency (e.g. means) or other basic estimates (e.g. regression coefficient) AND variation (e.g. standard deviation) or associated estimates of uncertainty (e.g. confidence intervals)
☒	☐	For null hypothesis testing, the test statistic (e.g. *F*, *t*, *r*) with confidence intervals, effect sizes, degrees of freedom and *P* value noted<br>*Give P values as exact values whenever suitable.*
☒	☐	For Bayesian analysis, information on the choice of priors and Markov chain Monte Carlo settings
☒	☐	For hierarchical and complex designs, identification of the appropriate level for tests and full reporting of outcomes
☐	☒	Estimates of effect sizes (e.g. Cohen's *d*, Pearson's *r*), indicating how they were calculated

Our web collection on statistics for biologists contains articles on many of the points above.

Software and code

Policy information about availability of computer code

Data collection	Practice Fusion EHR
Data analysis	No software was used

For manuscripts utilizing custom algorithms or software that are central to the research but not yet described in published literature, software must be made available to editors and reviewers. We strongly encourage code deposition in a community repository (e.g. GitHub). See the Nature Portfolio guidelines for submitting code & software for further information.

Data

Policy information about availability of data

All manuscripts must include a data availability statement. This statement should provide the following information, where applicable:

- Accession codes, unique identifiers, or web links for publicly available datasets
- A description of any restrictions on data availability
- For clinical datasets or third party data, please ensure that the statement adheres to our policy

Anonymized individual participant data and study documents can be requested for further research from www.clinicalstudydatarequest.com

Human research participants

Policy information about [studies involving human research participants and Sex and Gender in Research.](#)

Reporting on sex and gender	De-identified baseline characteristics, including sex (male only [n=5584]), were collected from the retrospective patient cohort. The patient cohort was also described by male sex (n=5584) stratified by asthma control status.
Population characteristics	Age, sex, ethnicity, race, smoking status, body mass index, healthcare provider visit type.
Recruitment	N/A
Ethics oversight	N/A

Note that full information on the approval of the study protocol must also be provided in the manuscript.

Field-specific reporting

Please select the one below that is the best fit for your research. If you are not sure, read the appropriate sections before making your selection.

☒ Life sciences ☐ Behavioural & social sciences ☐ Ecological, evolutionary & environmental sciences

For a reference copy of the document with all sections, see [nature.com/documents/nr-reporting-summary-flat.pdf](#)

Life sciences study design

All studies must disclose on these points even when the disclosure is negative.

Sample size	'A retrospective cohort was established which included patients with asthma and a valid ACT measurement in Practice Fusion's EMR database between January 1, 2015 and December 31, 2018, and with at least 1 prescription for any asthma treatment in the 6 months prior to the 4-week recall period of their first valid ACT measurement. The date of a patient's first valid ACT measurement was defined as their index date. Patients were required to have activity in the database, defined as an encounter in the database for any reason, at least 6 months (182 days) prior to their index date.'<br>'We identified 15,579 treated patients with asthma for our study sample after applying all inclusion and exclusion criteria'
Data exclusions	'Patients were excluded from our sample if they had ≥1 chronic obstructive pulmonary disease diagnosis code(s) reported at any time on or before their index date or had a missing value for their calendar year of birth'
Replication	NA: this was a retrospective observational study (HO-17-17251) of patients with asthma in the US, identified in Practice Fusion's EMR database.
Randomization	Patients were grouped by asthma control status, using the Asthma Control Test (ACT), and by GINA Step.<br>Asthma control status:<br>'ACT scores range from 5 (poor control of asthma) to 25 (complete control of asthma) with higher scores reflecting greater asthma control. ACT scores ≤19 reflect NWC asthma while ACT scores >19 reflect well-controlled (WC) asthma.13<br>Beginning in 2015, Practice Fusion implemented a clinical decision support program that notified providers that an ACT should be conducted when a patient with asthma missing symptom assessments visited them. While the notification indicated that an ACT should be completed, the system did not require clinicians to complete and/or record the ACT results.<br>We defined a valid ACT as: 1) having complete responses for all 5 questions; 2) not occurring on the same date as another ACT measurement for the same patient; and 3) not occurring within 28 days of another ACT measurement for the same patient. Scores that reflect asthma control as measured by the ACT cannot be calculated if any of the 5 questions are missing responses. The rationale behind this 28-day time gap is that the ACT reflects a 4-week recall period; if two ACT scores are measured on the same day or within 28 days of each other, it is impossible to determine which of these indicate the correct measurement of asthma control.'<br><br>GINA Step<br>'GINA Step was assessed based on the medications prescribed during the 6-month period prior to the 4-week recall period of patients' ACT record at index date. Asthma treatment was defined as one of the following medications: short-acting β2-agonists (SABA), short-acting muscarinic antagonist (SAMA), inhaled corticosteroids (ICS), ICS and long-acting β2-agonist (ICS/LABA) combination products, leukotriene receptor antagonist, cromolyn or nedocromil (mast cell stabilizers), methylxanthines, or long-acting muscarinic agonist (LAMA).'
Blinding	NA: this was a retrospective observational study (HO-17-17251) of patients with asthma in the US, identified in Practice Fusion's EMR database.

Reporting for specific materials, systems and methods

We require information from authors about some types of materials, experimental systems and methods used in many studies. Here, indicate whether each material, system or method listed is relevant to your study. If you are not sure if a list item applies to your research, read the appropriate section before selecting a response.

Materials & experimental systems

n/a	Involved in the study
☒	☐ Antibodies
☒	☐ Eukaryotic cell lines
☒	☐ Palaeontology and archaeology
☒	☐ Animals and other organisms
☒	☐ Clinical data
☒	☐ Dual use research of concern

Methods

n/a	Involved in the study
☒	☐ ChIP-seq
☒	☐ Flow cytometry
☒	☐ MRI-based neuroimaging
